# Toward Simplified Electrode Design: Development of Nickel-Efficient Catalytic NanoPTL for Sustainable AEM Water Electrolysis

**DOI:** 10.1021/acsnanoscienceau.5c00168

**Published:** 2026-03-18

**Authors:** Seungyoung Park, Seulgi Ji, Sara Andrenacci, Sun Sook Lee, Yejung Choi

**Affiliations:** † 34979 Kyonggi University, School of Electronic Engineering, Suwon 16227, Republic of Korea; ‡ Thin Film Research Center, 65680 Korea Research Institute of Chemical Technology, Yuseong, Post Office Box 107, Daejeon 34114, Republic of Korea; § Department of Sustainable Energy Technology, 275243 SINTEF Industry, 7034 Trondheim, Norway

**Keywords:** porous transport layer, bifunctional catalyst, nickel electrode, AEMWE, magnetically assisted chemical reduction

## Abstract

Nickel-based porous transport layers (PTLs) are widely used in anion exchange membrane water electrolysis (AEMWE) owing to their chemical stability in alkaline media, electrical conductivity, and efficient transport of gases and electrolyte. However, the rising cost and supply concerns of nickel necessitate more material-efficient solutions. This study introduces a freestanding, bifunctional catalytic Ni nanoPTL for AEMWE, synthesized via magnetically assisted chemical reduction. By tuning the nanowire morphology through seed concentration, the nanoPTL achieves up to 90% nickel reduction while enhancing the surface area and mechanical robustness. Electrochemical and structural analyses reveal strong morphology–activity correlations, with the nanoPTL outperforming commercial benchmarks in the hydrogen evolution reaction (HER) and showing promising oxygen evolution reaction (OER) performance. Single-cell tests confirm the stability and integration potential of the nanoPTL, underscoring both its potential and areas for improvement toward cost-effective, sustainable, and high performance AEMWE electrode design.

## Graphical abstract

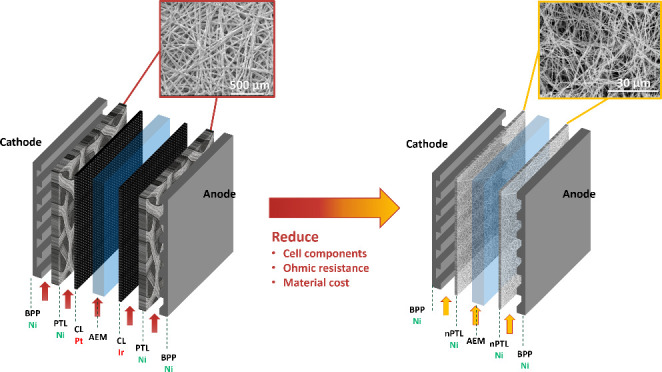

## 1 Introduction

In the era with ever-heightened environmental awareness and a global push toward decarbonization, hydrogen has emerged not only as a promising energy carrier but also as one of the most actively promoted solutions for a sustainable energy future. Its high energy density and potential for clean production have placed it at the forefront of sustainable energy strategies. Among the various hydrogen production methods, water electrolysis offers a direct and carbon-free pathway, particularly when it is powered by renewable electricity. Within this domain, anion exchange membrane water electrolysis (AEMWE) has recently attracted significant attention as a promising next-generation hydrogen production technology because it integrates the advantages of both traditional alkaline water electrolysis (AWE) and proton exchange membrane water electrolysis (PEMWE) while addressing many of their limitations. It combines the low OPEX of PEMWE and the low CAPEX of AWE technology.^1–4^ Compared to PEMWE, which relies on corrosive acidic environments requiring expensive noble metal catalysts such as Pt, Ir, and Ru, AEMWE operates under alkaline conditions that enable the use of earth-abundant and cost-effective catalysts (e.g., Ni, Fe, Co), thereby offering a clear pathway to reduce material and system costs for large-scale hydrogen production.^5,6^

Unlike conventional AWE, which uses liquid alkaline electrolytes and diaphragms with challenges including poor gas tightness and mass transport limitations, AEMWE employs a solid polymeric anion exchange membrane that physically separates the anode and cathode, significantly reducing gas crossover and allowing higher hydrogen purity and more compact cell designs. In addition, AEMWE shares many design advantages with PEMWE, such as the potential for zero-gap configurations and high current density operation while avoiding the high cost and limited catalyst choices imposed by acidic environments in PEM systems. These combined features such as low catalyst cost, improved gas separation, and favorable mass and charge transport characteristics explain why AEMWE has become a focal point in recent water electrolysis research aimed at producing green hydrogen economically from renewable energy sources.^6,7^

Despite its potential, AEMWE remains a relatively immature technology with substantial room for innovation in both cost reduction and performance, particularly in electrode design. While much research has focused on developing non-platinum group metal (PGM) catalysts for the oxygen and hydrogen evolution reactions (OER and HER), the performance gap between ex situ catalyst activity and in situ cell efficiency remains a challenge.^8–11^ This gap is often linked to limitations in catalyst layer architecture, ionomer distribution, and the structure of the porous transport layer (PTL).

Nickel is commonly used in PTLs and catalysts for AEMWE owing to its favorable electrical conductivity, corrosion resistance, and intrinsic catalytic activity in alkaline environments.^12,13^ Its relatively low cost and high abundance have positioned it as a preferred alternative to PGM. However, recent geopolitical instability has led to significant nickel price volatility and supply concerns, driving the demand for nickel-efficient electrodes. To address these challenges, we build upon previously reported nickel nanowire foam synthesis methods and present an optimized, freestanding catalytic nanoPTL synthesized via a magnetically assisted chemical reduction process.^14,15^ While earlier studies have demonstrated the potential of such nanowire structures as freestanding HER and OER electrodes in three-electrode configurations, the nanowire morphology tuning and their application as integrated porous transport layers in full AEMWE cells have not yet been explored.

In this work, we identify the optimal concentration of silver (Ag) and platinum (Pt) as heterogeneous seed elements to control the nanowire morphology to form a highly active, mechanically robust, and multiporous structure suitable for direct integration into the cell. This bifunctional nanoPTL simplifies the cell configuration by combining catalyst and transport layer functions, eliminates the need for ionomer complications and catalyst delamination risk, and achieves up to 90% reduction in nickel usage compared with commercial PTLs. To systematically understand the correlation between synthesis parameters and material performance, we investigate the structural, chemical, and electrochemical characteristics of the nanoPTLs to understand how synthesis parameters influence material performance. Finally, we validate the practical applicability of the catalytic nanoPTL in a preliminary single cell, demonstrating its integration potential and highlighting its promise for scalable, cost-effective, and sustainable AEMWE electrode design.

## 2 Materials and Methods

### 2.1 Synthesis of Catalytic NanoPTL

The Ni catalytic nanoPTL was fabricated by hydrazine assisted chemical reduction at ambient temperature under a magnetic field, following the literature with some modifications.^16,17^ The experimental schematic is illustrated in Figure 1a. Briefly, two separate solutions were prepared, one containing 2.25 mmol of sodium citrate, 6 mmol of nickel chloride, and 0.024 mmol of silver nitrate (0.4 mol % to Ni) in 30 mL of DI water, and the other containing 30 mL of aqueous solution with 8 vol % hydrazine hydrate. Both solutions were pH regulated to 12.5 by adding NaOH solution and then preheated up to 80 °C in an oil bath. Subsequently, the two solutions were combined and left in the oil bath for 1 h. A magnetic field was applied to the solution mixture in a directionally discontinuous manner during the growth period by placing a magnet near the surface of the solution and horizontally spinning the magnet at 30 rpm using an overhead stirrer. The stirring of the magnet was conducted to prevent unidirectional growth of the wires and instead promote random intertwined growth of the wires for better interconnection. After an hour of reaction, the reaction medium turned colorless and clear, indicating complete consumption of Ni ions, and yielded a cylindrical foam-like nanoPTL floating on the surface of the solution. Finally, the nanoPTL was washed several times with DI water and ethanol, followed by compression with a glass rod into a thin sheet and drying in a vacuum oven at 60 °C. The average areal mass of nanoPTL is 15 mg cm^–2^.

**Figure 1:**
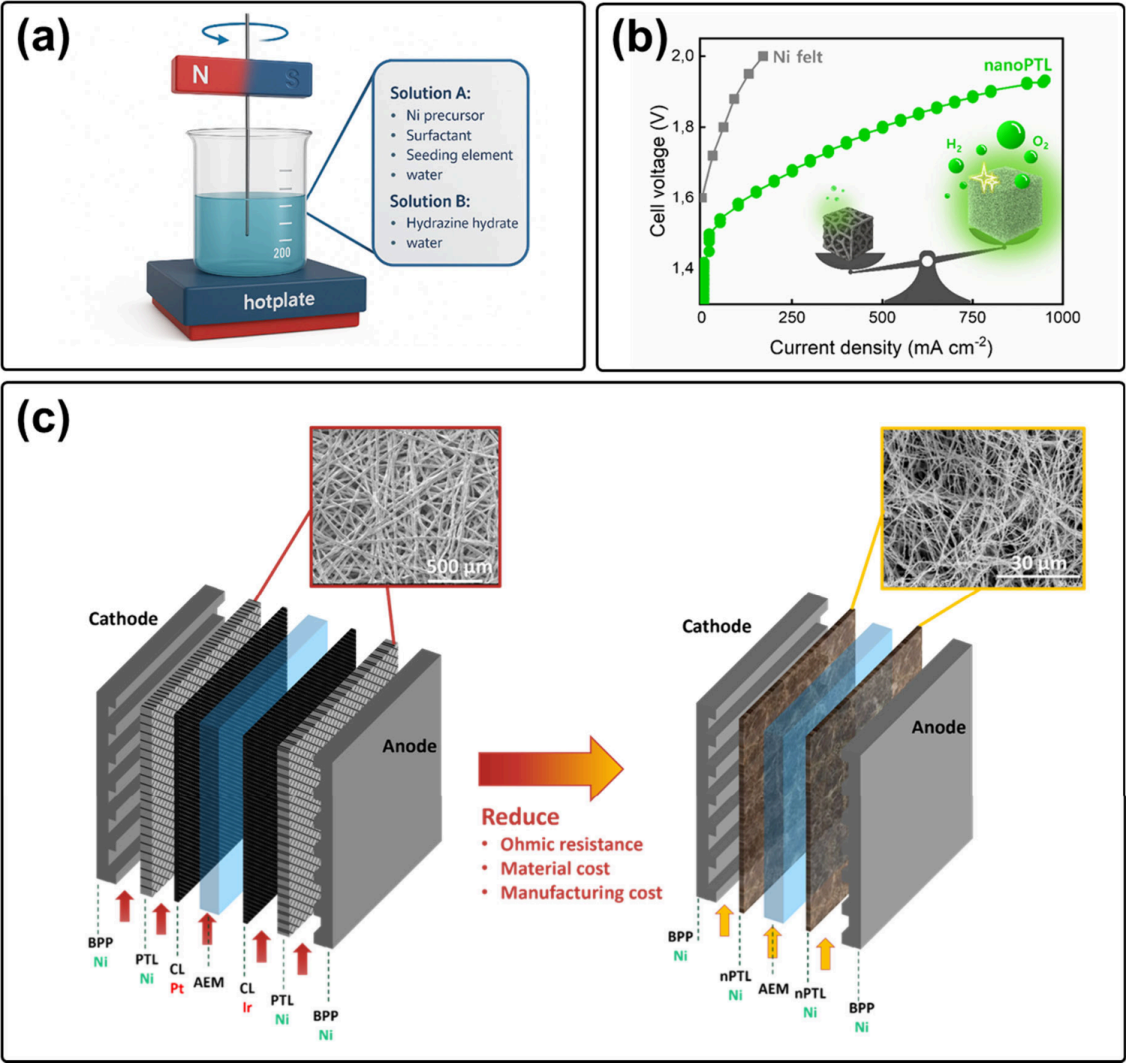
(a) Schematic diagram of the synthesis setup, (b) overall concept of nanoPTL, and (c) proposed simplified cell configuration using nanoPTL.

### 2.2 Ex Situ Three-Electrode Electrochemical Test

The three-electrode system tests were carried out on a Zive MP4 electrochemical workstation (WonATech) at room temperature. A piece of nanoPTL (1 cm^2^) served as the working electrode, while a graphite rod and a Hg/HgO electrode were used as the counter electrode and reference electrode, respectively, in 1 M KOH electrolyte. Linear sweep voltammetry (LSV) tests were used to evaluate the activity of the electrocatalysts for the HER and the OER at a scan rate of 1 mV s^–1^. All tested electric potentials were expressed in the reversible hydrogen electrode (RHE) scale according to the Nernst equation: *E*_RHE_ = *E*_Hg/HgO_ + 0.14 V + 0.059 × pH. All LSV curves were compensated for ohmic losses using 100% *iR* correction based on the high frequency resistance obtained from electrochemical impedance spectroscopy (EIS). Potentiostatic electrochemical impedance spectroscopy (EIS) was carried out in frequencies ranging from 100 kHz to 1 Hz at −0.2 V vs RHE for HER and 1.8 V vs RHE for the OER. 10 mV was applied as the amplitude. Stability measurements of the nanoPTLs were conducted at a constant current hold at 1 A cm^–2^ for the HER and a constant potential hold at 1.75 V for the OER.

### 2.3 In Situ Single-Cell Test

The nanoPTLs were assembled with a commercial membrane (cannot be disclosed due to material transfer agreements) in homemade 6.25 cm^2^ nickel single-cell hardware. Commercial Ni PTL for comparison was purchased from Bekaert. A catalyst coated substrate (CCS) was also prepared by spraying commercial catalyst ink (Pt/C (also written as PtC), IrO_2_, 10 wt % ionomer) onto Ni felt (Bekipor2Ni, 0.5 mm, Bekaert) for the anode and Toray carbon paper (200 μm) for the cathode. The areal mass of Ni felt was 150 mg cm^–2^. The homemade single cell consisted of Ni flow fields, a Ni current collector, and stainless steel compression plates with bolts tightened with a torque wrench. In addition, the gasket thickness (Icecube from Freudenberg Performance Materials) was chosen to match the PTL thickness for thorough compression. The cell was operated at 1 M KOH, 60 °C, atmospheric pressure, with a flow rate of 30 mLPM. Polarization curves and EIS data were collected using an SP-150 potentiostat and a 20 A booster (Bio-Logic Science Instruments) under galvanostatic conditions with a cutoff voltage of 2 V. Polarization data were collected at 1 min per point, where the current was increased stepwise at a rate of 15 mA cm^–2^ up to 0.1 A cm^–2^, after which the rate was increased to 50 mA cm^–2^ up to 1 A cm^–2^, after which the rate was increased to 100 mA cm^–2^ up to the cutoff voltage. The EIS measurements were performed under galvanostatic conditions at 720 mA cm^–2^ over a frequency range of 100 kHz to 1 Hz, employing an AC amplitude of 36 mA cm^–2^. For the short-term stability test, the cell was operated at a constant current of 200 mA cm^–2^ for 20 h.

## 3 Results and Discussion

### 3.1 Structural and Chemical Characterization

Figure 1a illustrates a general schematic of the experimental setup. During the synthesis reaction, silver ions are primarily reduced upon the introduction of hydrazine to form silver seed particles before nickel reacts, owing to the relatively high reduction potential of silver. This phenomenon can be physically observed from the immediate color change of the reaction solution from clear green to dark gray upon the addition of hydrazine solution in the presence of silver, as opposed to the slow color transition from green to blue over an hour in the absence of silver. Thus, silver particles serve as heterogeneous nuclei for the subsequent growth of nickel particles. It is a common synthetic strategy to use some sort of a heterogeneous seeding method to accelerate the growth step and control the particle size and uniformity. The seeds facilitate nucleation of the metals of interest by providing the preferential sites for nucleation with low effective surface energy, thus diminishing the free energy barrier for nucleation. The seed assisted reaction rate acceleration can be witnessed from the significantly faster rate of Ni ion consumption in the presence of silver, resulting in a large metallic foam and colorless clear solution after an hour (Figure S1).

Silver seeds were chosen to derive Ni nanoPTL formation, as opposed to the previously reported and more commonly used method that involves platinum seeds. Considering the cost and scarcity of platinum that limit its long-term large-scale usage, substitution with a silver reagent was attempted for its low cost, abundance, and comparably high reactivity. Moreover, the fact that the high reduction potential of silver (Ag^+^/Ag: 0.80 V) exceeds that of platinum, which takes place in two steps (PtCl_6_^2–^/ PtCl_4_^2–^: 0.68 V; PtCl_4_^2–^/Pt 0.73 V), also makes this substitution a logical choice.^18^ To test the feasibility of this substitution, reactors containing increasing amounts of platinum seed solution and an equivalent amount of silver seed solution were allowed to react for 1 h and then compared. Figure S1 intuitively illustrates that reactions seeded with Ag proceed more rapidly and consume reactants more effectively than those with Pt, as evident from the color change of the solution. While the Pt-seeded reactors retain a blue hue, indicating the presence of unreacted Ni precursor ions, the Ag-seeded reactor appears colorless, suggesting a complete consumption of reactants within the same time frame.

The reactions involved in the synthesis can be simply expressed in the equations below:

1 $$N_{2}H_{4}+4{\mathrm{OH}}^{-}\to N_{2}+H_{2}O+4e^{-}$$

2 $$N_{2}H_{4}+{\mathrm{OH}}^{-}\to N_{2}+{\mathrm{NH}}_{3}+H_{2}O+e^{-}$$

3 $$N_{2}H_{4}+4{\mathrm{Ag}}^{+}+4{\mathrm{OH}}^{-}\to N_{2}+4\mathrm{Ag}+4H_{2}O$$

4 $$\mathrm{Ni}\left(\mathrm{II}\right)+2e^{-}\to \mathrm{Ni}$$

During the formation of Ni nanoPTL, one can observe vigorous gas evolution within the reaction medium, whereas no significant amount of gas evolution is observed in the absence of hydrazine. The gas evolution is due to the catalytic decomposition of hydrazine into hydrogen and nitrogen gas.^19^

5 $$N_{2}H_{5}\mathrm{OH}\to 2H_{2}+N_{2}+H_{2}O$$

The arrangement of Ag-seeded Ni nanoparticles into wires is promoted by the applied magnetic field and the intrinsic ferromagnetic properties of metallic nickel. The Ni species are assembled onto the spiky particles with the same direction as the magnetic domain and grow into nanowires. Furthermore, shifting the direction of the applied magnetic field via slow spinning enables intertwined alignment of the wires into a flexible foam, which is a Ni nanoporous transport layer (nanoPTL) with good mechanical strength. Fine-tuning of the wire thickness is possible by controlling the amount of silver nitrate introduced into the reaction system. Assuming that at a given temperature, the size of the Ag nuclei is independent of the added molar concentration of silver (mol %_Ag_) and that each Ag nucleus yields a single nickel particle, the number of the final metal particles would be directly proportional to mol %_Ag_, while the size of the final metal particles would be inversely proportional to mol %_Ag_. This relationship has been theoretically and experimentally investigated and proven in previous literature works and this study as well.^20,21^ A decreasing trend in wire thickness has been observed via SEM and TEM as mol %_Ag_ increased up to 0.4% (Table S1, Figures S2 and S3). However, when the molar content of Ag exceeded 0.4% of Ni, the wire thickness deviated from the trend, and the polydisperse formation of thick and chunky strands was observed. This can be attributed to the generation of an excessive number of silver nuclei within the medium that lead to coagulation of the nuclei, resulting in larger polydisperse metal particles forming the wire. A less evident but similar trend is observed in the growth of platinum-seeded nanowires, indicating that the seed metal salt concentration affects the nanowire thickness (Table S1, Figure S4).

While three-electrode electrochemical evaluations provide valuable insights into the intrinsic activity and stability of individual electrode materials, the observed characteristics rarely translate directly into full-cell performance. Factors such as electrode–electrolyte interface behavior, gas evolution dynamics, and cell architecture can significantly influence the operational characteristics in a single-cell setup. To validate the practical applicability of the nanoPTL, assess its performance under realistic conditions, and identify improvement points for further studies, a series of single-cell tests using a matrix of five anode/cathode combinations were conducted. Figure 1b illustrates the overall concept of the nanoPTL designed to be lighter, Ni-efficient, and still superior in performance through nanostructuring. Figure 1c shows a graphical representation of the cell configurations used in the testing series.

Figure 2a shows an individual nanowire with a spiky surface, which constitutes the Ag-seeded nanoPTL. In order to identify the influence of the seed element, further chemical and morphological characterizations of the nanoPTLs were carried out. The XRD patterns of the Ag- and Pt-seeded nanoPTLs with different Ag and Pt concentrations are recorded in Figure 2b. The three well-defined diffraction peaks at 44.51°, 51.91°, and 76.41° for all four samples are indexed to the typical (111), (200), and (220) planes of face-centered cubic (fcc) nickel (JCPDS No. 04-0850). A slight shift toward lower angles can be observed for the Pt 0.4% sample, while no obvious peak from Ag or Pt can be observed from the XRD spectra. The lower angle shift observed in Pt-seeded Ni indicates Ni lattice expansion due to Pt incorporation, while the fcc structure of Ni is preserved.^22^ Ag-seeded Ni does not exhibit lattice expansion, likely due to the greater lattice mismatch, lower surface energy of Ag, and immiscibility between Ag and Ni.^23,24^ It has been confirmed that Ag–Ni is the most thermodynamically stable in the form of a core–shell structure.^25^

**Figure 2:**
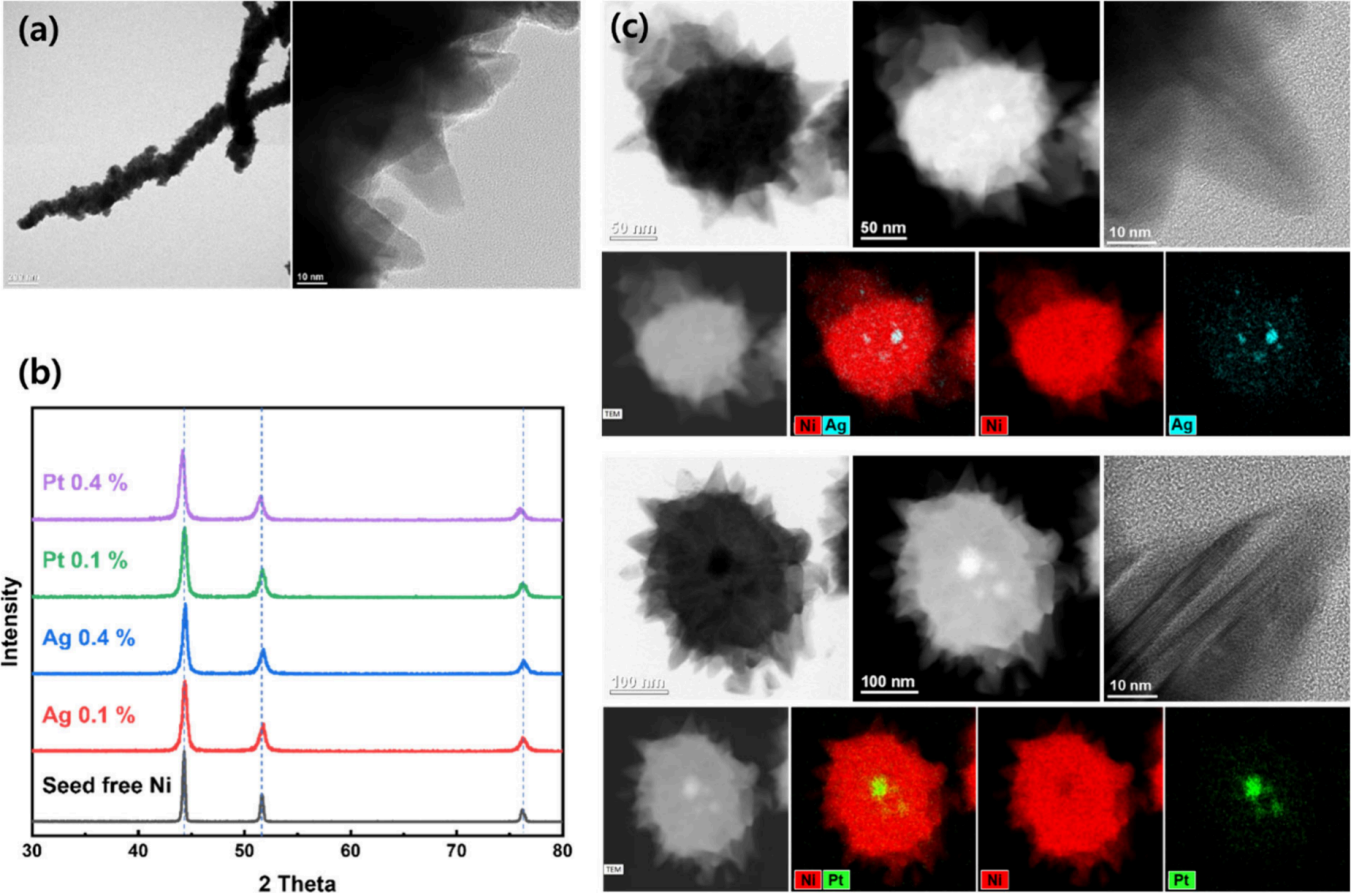
(a) TEM images of an individual nanowire with the spiky shape of the nanoPTL with Ag seeds. (b) XRD patterns of nanoPTLs synthesized with Ag and Pt at 0.1% and 0.4%, showing characteristic Ni (111), (200), and (220) peaks. (c) Cross-sectional TEM images and TEM-EDS mapping images of (top) Ag-seeded and (bottom) Pt-seeded nanoPTLs.

To clarify the composition and the structure of individual nanowires forming the nanoPTL, cross-sectional TEM analysis and TEM-EDS mapping of Ag- and Pt-seeded nanowires were performed (Figure 2c). The mapping images indicate that Pt and Ag are largely present within the core wrapped with a spiky nickel shell, forming a core–shell structure. The HR-TEM images shown in Figure S5 confirm that the lattice distance of the spiky sheets on the surface corresponds to the Ni (111) plane. Transient core–shell growth was monitored with HR-TEM at 10 and 30 min, and both seeding methods showed evidence of growth in core–shell form (Figures S6–S9).

The core–shell structure and elemental composition of the nanoPTL were further confirmed from XPS depth profiling, where both Ag and Pt cores were detected only after some etching rounds (Figure S10). The Ni 2p spectrum exhibits characteristic peaks at 855.5 eV (Ni 2p_3/2_) and 873.5 eV (Ni 2p_1/2_). Only the first layer shows satellite peaks representing Ni^2+^ species, which disappear after etching rounds, suggesting the presence of nickel oxide or hydroxide at the surface. For Ag, the Ag 3d_5/2_ peak appears at ∼368.2 eV with increasing etching time, indicating the presence of metallic silver near the core. In the Pt-seeded sample, the Pt 4f_7/2_ and 4f_752_ peaks are observed at ∼71.2 and at ∼74.5 eV as etching time increases, indicating the presence of a metallic Pt subsurface. The O 1s shows two main peaks at ∼529.0 and ∼531.5–532.0 eV, attributed to metal oxides and hydroxide species or adsorbed water, respectively. The dominant metal hydroxide peak confirms the presence of nickel hydroxides on the surface of the nanoPTL. Depth profiling via XPS reveals a gradual increase in Ag and Pt atomic ratios with etching time, confirming their presence under the Ni shell with a hydroxide rich surface (Figure S10). The exposed nickel shells were free of seed elements in both seeding methods, indicating no difference in chemical composition, once again confirming the identical Ni lattice distance observed from HR-TEM of the spiky surfaces. Additionally, BET analysis was performed as the physical characterization to observe the difference in surface area of the Ag- and Pt-seeded nanoPTLs. The Ag-seeded nanoPTL showed an increase in surface area from 2.5542 to 3.1224 m^2^/g as the Ag concentration increased from 0.1% to 0.4%, which is a 56% larger value compared to that of the Pt 0.4% nanoPTL (Figure S11, Table S2).

### 3.2 Electrochemical Properties of Catalytic NanoPTL

The electrocatalytic performances of catalytic nanoPTLs with varying seed concentrations were evaluated in 1.0 M KOH using a typical three-electrode electrochemical setup. As shown in Figure 3A, for HER LSV, all nanoPTL samples significantly outperformed not only the commercial Ni felt but also PtC/GDL at high current density, demonstrating the catalytic viability of the nanowire-based architecture. Among the nanoPTLs, HER activity improved with increasing Ag seed concentration up to 0.4%, beyond which the performance declined. This trend matches the nanowire morphology, particularly the wire thickness and surface area, and suggests a strong structure–activity correlation. Ag 0.4% nanoPTL shows a remarkably high current density of nearly 1900 mA cm^–2^ at −0.22 V vs RHE, which is a 6-fold higher performance than that of PtC/GDL at the same applied potential.

**Figure 3:**
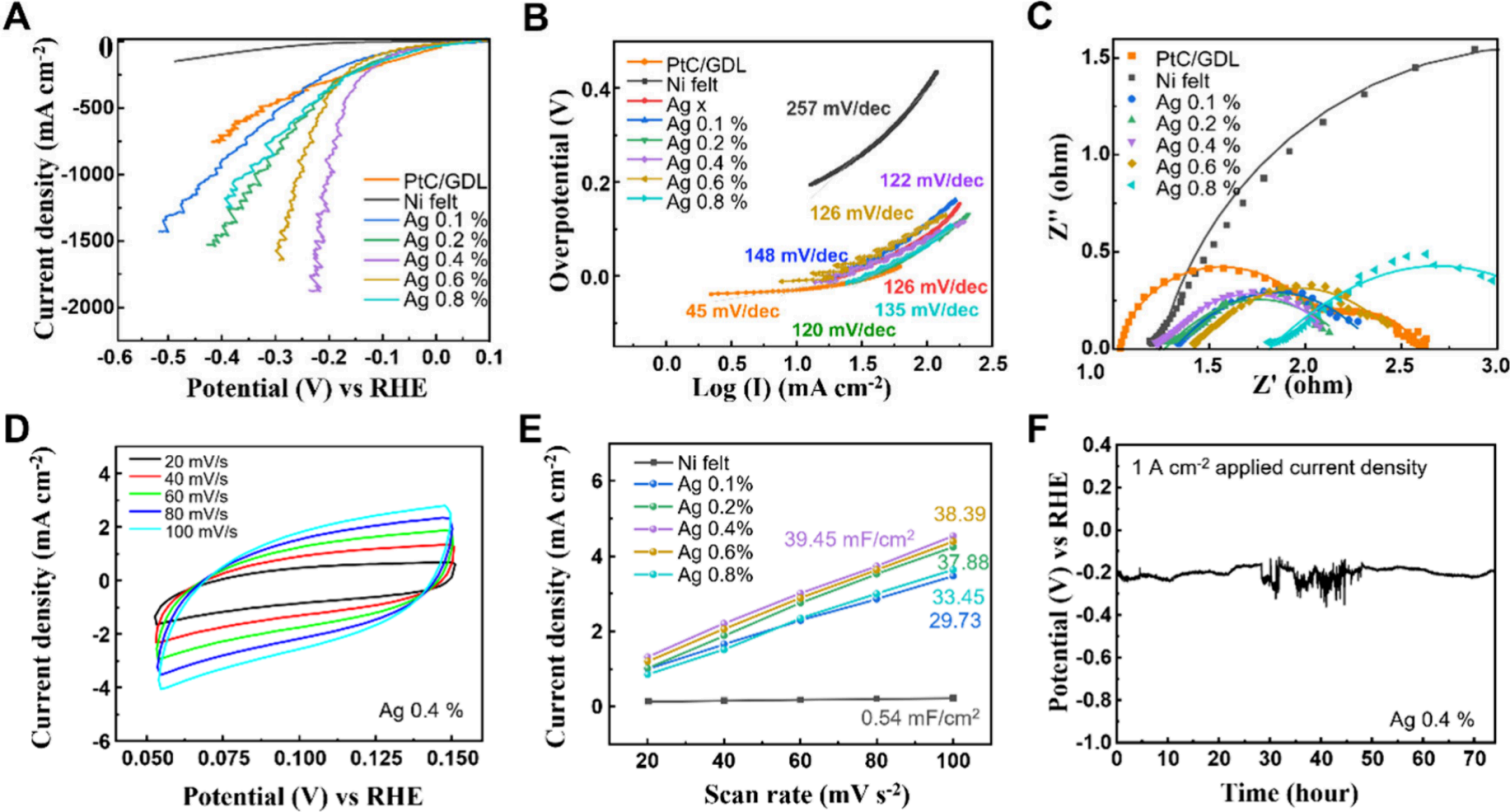
Electrochemical analysis of catalytic nanoPTL for HER: (A) LSV curves, (B) Tafel plots, (C) EIS Nyquist plots, (D) CVs scanned at different scan rates, (E) double layer capacitance plots, and (F) chronopotentiometry stability test conducted on the best-performing Ag 0.4% sample for over 70 h.

The Tafel slopes in Figure 3B hint that the observed performance is largely related to structure and surface area rather than intrinsic kinetics, as the Ag 0.4% nanoPTL exhibits a much larger slope (122 mV/dec) than PtC/GDL (45 mV/dec). Moreover, all nanoPTL samples exhibit similar Tafel slopes (ranging from ∼120 to 148 mV/dec), indicating that the intrinsic HER kinetics are largely unaffected by seed concentration. This suggests that the observed performance differences are primarily due to morphological factors such as surface area and pore structure, rather than variations in reaction mechanism or catalytic activity. Note that the commercial Ni felt displays a larger Tafel slope, which may be attributed to its less favorable surface chemistry. The M–OH peak in the XPS spectra and the wire surface covered in a spiky sheet-like morphology observed in the SEM analyses suggest that nanoPTLs possess a rich distribution of catalytically active nickel hydroxides that are likely to enhance HER activity better than the smooth, thick microfibers in Ni felt.

Electrochemical impedance spectroscopy (EIS) measurements were conducted to assess charge transfer characteristics across the different nanoPTL samples in a qualitative context, as shown in Figure 3C. Notably, the conventional nickel felt exhibits a slightly higher charge transfer resistance compared to the Ag-seeded nanoPTLs, which can be attributed to its relatively limited electrochemically active surface area and less effective electrode–electrolyte interfacial contact. In contrast, the Ag-seeded nanoPTLs provide a more uniformly distributed nanoscale morphology, leading to enhanced interfacial contact and more efficient charge transfer kinetics. The overall trends suggest similar charge transfer resistance among the Ag-seeded nanoPTLs and further support the conclusion that performance differences are closely tied to morphology and surface area. Double layer capacitance measurements further support the HER performance–nanoPTL morphology correlation in Figure 3D,E. While the *C*_dl_ values among nanoPTL samples are relatively close but still follow the order of the HER performance from the LSV plots, they are significantly higher than that of commercial Ni felt, reflecting the enhanced electrochemically active surface area provided by the nanowire architecture.

To evaluate the electrochemical stability of the nanoPTL, a 74 h chronopotentiometry test was conducted on the best-performing Ag 0.4% sample. As shown in Figure 3F, the potential remains relatively stable over the continuous operation, with no significant increase in overpotential. The large disturbance between 30 and 50 h was due to graphite counter rod dissolution and electrolyte contamination. The signal smoothed out again when the electrolyte was replaced with fresh electrolyte. The sample did not show any signs of physical damage after over 70 h of vigorous gas evolution at 1 A cm^–2^, suggesting the excellent electrochemical durability and structural integrity of the nanowire architecture.

Based on the established theory that nanoPTLs exhibit uniform surface chemistry and that the observed HER performance is primarily governed by morphology and surface area rather than kinetics, OER characterization was conducted only on the best-performing Ag 0.4% nanoPTL sample to maintain a focused and resource-efficient approach. This strategy enables the assessment of the full water-splitting capability of the most effective nanoPTL design while minimizing efforts on less promising variants. For clarity, the best-performing Ag 0.4% sample is hereafter referred to as “nanoPTL”. As shown in Figure 4, the OER characteristics of the nanoPTL sample were evaluated alongside the commercial Ni felt and IrO_2_/Ni felt benchmark. In the polarization curves (Figure 4a), nanoPTL exhibits a significantly higher current density at a given overpotential compared to the bare Ni felt but still a lower performance than the IrO_2_/Ni felt. This indicates clear contribution from the nanowire architecture while showing room for improvement in intrinsic catalytic efficiency.

**Figure 4:**
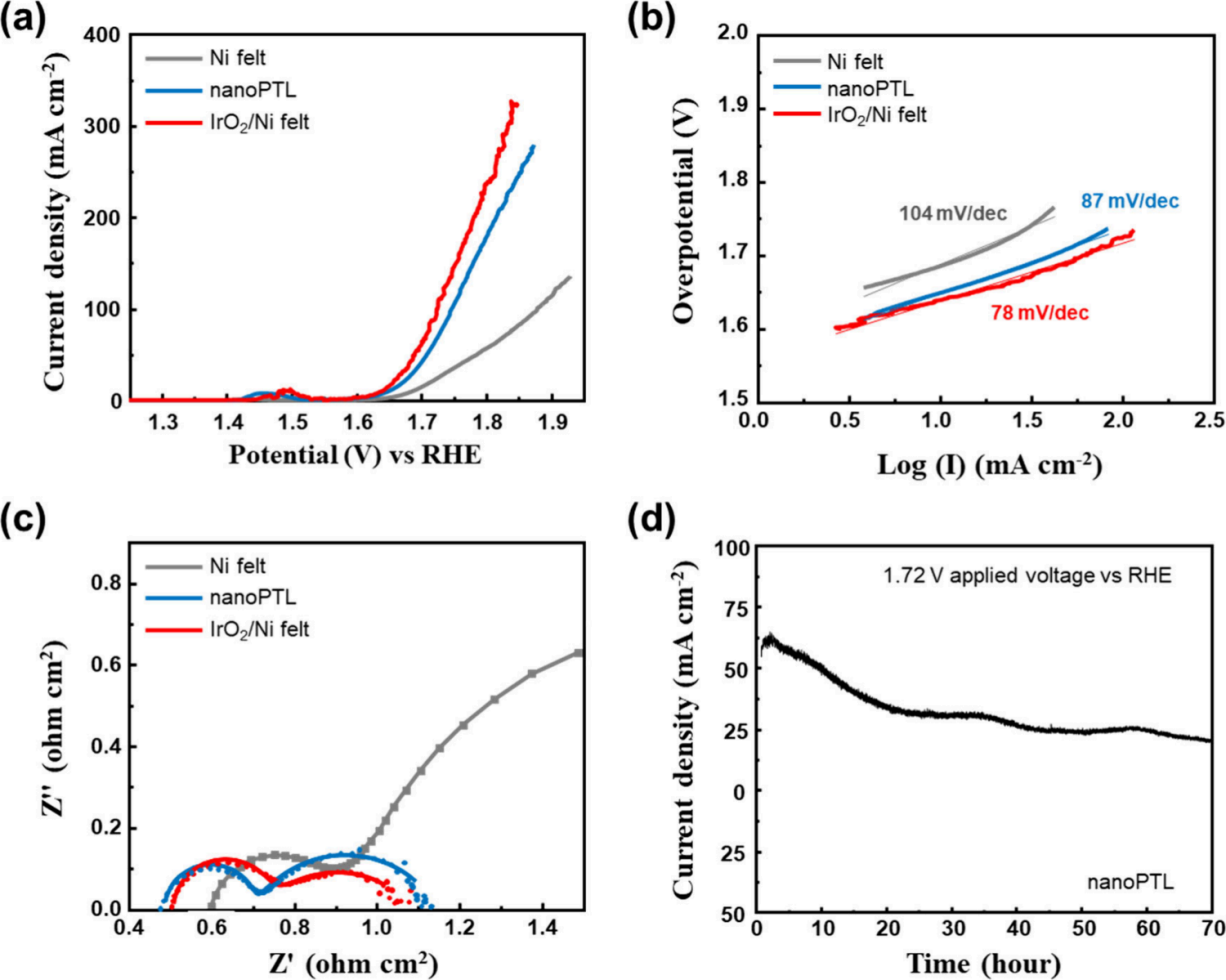
Electrochemical analysis of catalytic nanoPTL for the OER: (a) LSV curves, (b) Tafel plots, (c) EIS Nyquist plots, and (d) chronopotentiometry stability test with the best-performing Ag 0.4% sample.

The Tafel plots mirror the trend observed in LSV, with consistent reaction kinetics in Figure 4b. The bare Ni felt shows a slope of 104 mV/dec. NanoPTL shows a value of 87 mV/dec, and the IrO_2_/Ni felt shows a value of 78 mV/dec. EIS also indicates that nanoPTL exhibits a lower *R*_CT_ than bare Ni felt, though this value is still higher than that of IrO_2_/Ni felt in Figure 4c. Chronoamperometric measurements of the nanoPTL sample show a steep performance decay during the first 20 h, followed by a more gradual and steady decline in Figure 4d. Note that since the chronoamperometry is conducted potentiostatically, the resulting current response at the applied potential cannot be directly compared to the LSV values because *iR* correction is not applied during the chronoamperometric measurement. The initial drop is likely due to oxidation at the contact points of the nanowires, which reduces electrical conductivity. These results demonstrate that while nanoPTL possesses promising structural properties for the OER as well, further optimization is needed to mitigate conductivity losses from surface oxidation and to enhance its intrinsic catalytic activity toward the OER in order for it to compete with the commercial benchmark.

Nickel-based electrodes operated in alkaline media are known to incorporate trace Fe impurities from the electrolyte and exhibit enhanced OER activity after extended cycling or conditioning between potentials below the OER onset and the higher anodic potentials.^26,27^ It should be noted that the electrochemical protocols used in the present work including the short-term stability test are neither of sufficient duration nor within the potential window required to enable meaningful Fe incorporation. Moreover, the OER chronoamperometric response of the nanoPTL shows a steep decline attributed to degradation behavior rather than the characteristic activity improvement typically associated with Fe uptake. This behavior suggests that Fe incorporation is negligible under the conditions applied and that the observed degradation is instead dominated by nanowire oxidation and consecutive growth in contact resistance.

### 3.3 In Situ Single-Cell Characterization

While three-electrode electrochemical evaluations provide valuable insights into the intrinsic activity and stability of individual electrode materials, the observed characteristics rarely translate directly into full-cell performance. Factors such as electrode–electrolyte interface behavior, gas evolution dynamics, and cell architecture can significantly influence the operational characteristics in a single-cell setup. To validate the practical applicability of the nanoPTL, assess its performance under realistic conditions, and identify improvement points for further studies, a series of single-cell tests were performed using a matrix of five anode/cathode combinations. Figure 1c shows a graphical representation of the cell configurations used in the testing series.

Figure 5 presents the polarization curves of the full-cell configurations, each combining nanoPTL and commercial CCS as the anode and cathode electrodes to evaluate the performance of nanoPTL in a full-cell AEM electrolyzer setup. The written order of the legend represents the configuration as “cathode-anode”. The benchmark cell Pt/C-IrO_2_ as the anode shows high performance, reaching above 2 A cm^–2^ under 2 V, as expected due to the superior intrinsic activity of both commercial catalysts, while the Ni felt-Ni felt cell shows the poorest performance of merely 0.2 A cm^–2^ at 2 V. Although Ni felt can exhibit intrinsic electrochemical activity under certain conditions, it is most commonly utilized as a transport layer in realistic electrolyzer configurations, where unmodified Ni felt generally provides a limited catalytic contribution compared to purpose-designed catalytic electrodes.

**Figure 5:**
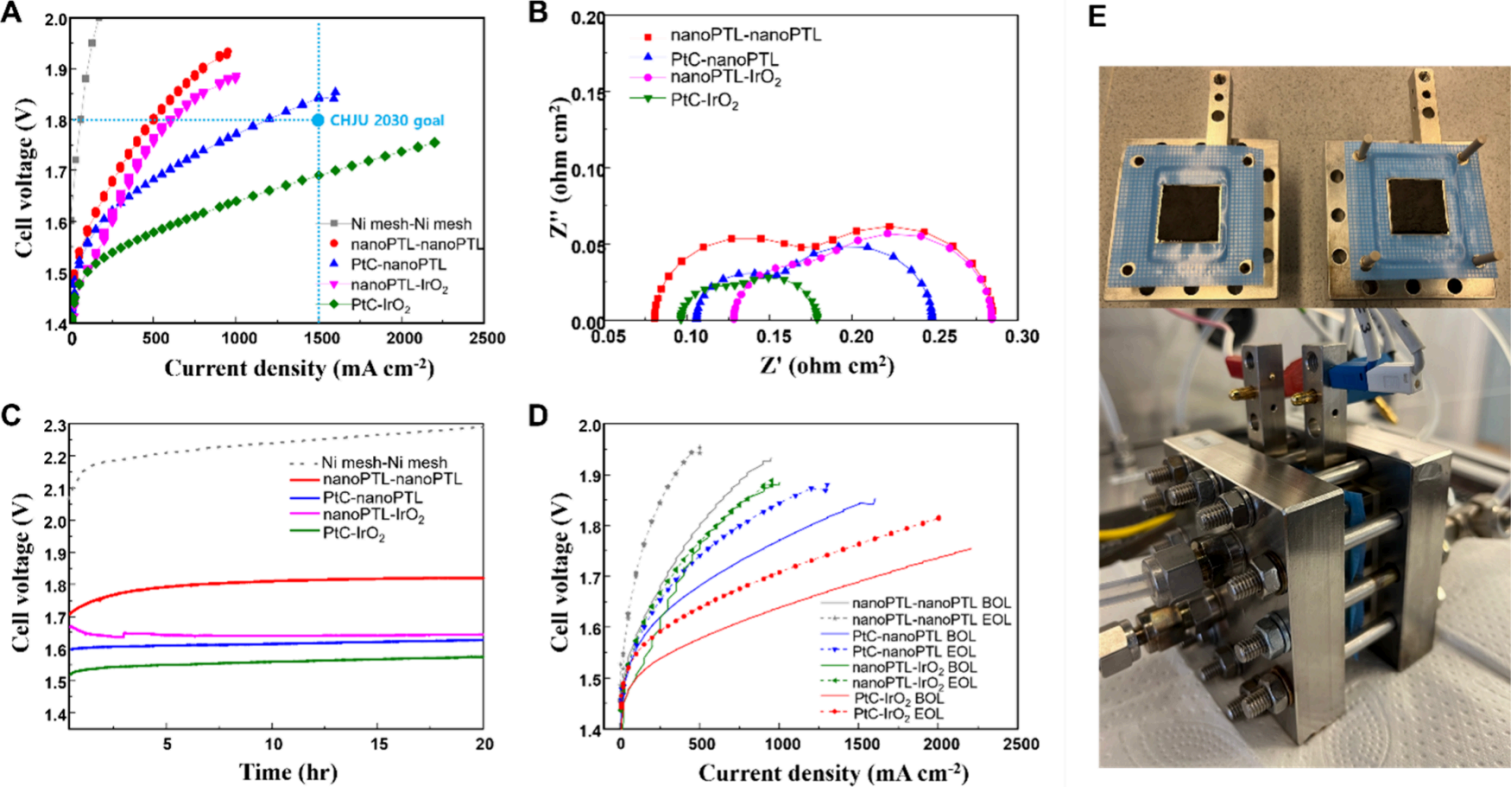
Single-cell characterization of nanoPTL electrodes in various combinations: (A) polarization curves, (B) galvanostatic EIS Nyquist plots, (C) 20 h short-term stability test at a constant current hold at 200 mA cm^–2^, (D) BOL vs EOL polarization curves, and (E) cell configuration and hardware.

The nanoPTL-nanoPTL cell demonstrates a large jump in current density compared to the Ni felt-Ni felt cell, showing 0.9 A cm^–2^ at 2 V. This value well exceeds the 2024 CHJU target for AEMEL (0.6 A cm^–2^ at approximately 2 V, 0.4 mg_CRM_/W) and is a good place to start optimization to reach the 2030 CHJU target (1.5 A cm^–2^ at approximately 1.8 V, 0 mg_CRM_/W).^28^ While it is a big jump from the Ni felt-Ni felt cell, it is still a far lower performance than the benchmark cell when compared to the three-electrode performance, which was better than the benchmark in the HER and only moderately behind that in the OER.

The Pt/C-nanoPTL cell shows improved performance compared to the full nanoPTL cell, highlighting the contribution of Pt/C to the HER efficiency. Compared to the benchmark, the polarization curve shows an overall shift to higher cell voltages, indicating the overall lower kinetics in nanoPTL than IrO_2_ as observed in the three-electrode tests. Conversely, the nanoPTL-IrO_2_ cell shows excellent kinetics that overlap with the benchmark polarization curve at low current densities, suggesting that nanoPTL provides just as good kinetics as Pt/C at low currents. However, beyond the low current density region, the polarization curve shows a notable shift to higher cell potentials and shows overlapping behavior with the full nanoPTL cell. This strongly insinuates that there are mass transport inefficiencies present within the nanoPTL owing to the more compressible nanoPTL structure compared to GDL, particularly when used as a cathode in single cells.

This interpretation is supported by the EIS analysis in Figure 5B, where the second semicircle, which is commonly associated with the mass transport process, of the nanoPTL-nanoPTL cell is nearly identical in size to that of nanoPTL-IrO_2_ cell. While the Nyquist plot provides insight into mass transport and charge transfer behavior, the high frequency resistance (HFR) was not used for comparative interpretation in this study. This is because nanoPTLs produced via small-scale synthesis exhibit unavoidable thickness variations, which alter cell compression during assembly. Since cell compression has a strong influence on the measured HFR, the resulting values are not a reliable comparative metric of intrinsic ohmic resistance between configurations.

To more systematically quantify loss contributions, voltage breakdown analysis (VBA) was conducted to deconvolute the total overpotential into ohmic, kinetic, and residual overpotentials (Figure S12).^29^ The VBA shows that the nanoPTL-IrO_2_ cell exhibits kinetic overpotentials comparable to those of the benchmark Pt/C-IrO_2_ cell, consistent with the overlapping low current density performance observed in Figure 5A. Moreover, the large residual overpotential that includes catalyst layer resistance, mass transport limitations, and other losses indicates that transport related limitations within the nanoPTL structure are the dominant performance penalty when it operates as the cathode.

A short 20 h stability test was carried out for each of the cells to evaluate the stability of the nanoPTL in a single cell. The nanoPTL full cell and Pt/C-nanoPTL showed degradation rates similar to that of the Pt/C-IrO_2_ benchmark, while the nanoPTL-IrO_2_ cell showed improvement in the beginning, followed by a steady voltage response showing no signs of degradation over 20 h. This indicates that nanoPTL does not degrade when used as the cathode and, in fact, may go through an activation process in the first 3 h. Polarization curves were collected after the stability test (end of life, EOL) to compare with the beginning of life (BOL) performance. While all the other cell combinations including the benchmark cell showed a large decline in performance, the unchanged current response of the nanoPTL-IrO_2_ cell at high current density further confirms the steady performance of nanoPTL as a cathode. The potential shift at low current density is speculated to come from the delamination or degradation of IrO_2_, judging from the similar kinetics of the benchmark EOL curve. However, the fact that the BOL and EOL polarization curves of the Pt/C-IrO_2_ cell do not overlap in the higher current density area highlights that there is more than one type of degradation taking place in the Pt/C-IrO_2_ cell.

Finally, the cells were disassembled and inspected for visible signs of physical distortion, delamination, or transfer of electrode materials onto the membrane (Figure 6). The benchmark cell showed severe blackening of the membrane in the active area due to IrO_2_ delamination from the electrode and deposition onto the membrane, leaving the Ni felt seemingly bare. Catalyst delamination and transfer/infiltration into the membrane are common issues in AEMEL due to the complexity of ionomer content tuning and iridium instability in alkaline media. The nanoPTL-IrO_2_ cell showed slightly better IrO_2_ retainment than the benchmark but still showed that IrO_2_ transferred onto the membrane. This may be due to the mechanical difference between the rigid, hard, planar, and relatively incompressible nature of Pt/C on GDL and the soft, compressible, and deformable nature of nanoPTL with relatively varying through plane thickness. The GDL provides uniform support to the membrane to press against the IrO_2_ layer during operation, which can promote full transfer of the catalyst layer if the adhesion of the catalyst layer to the substrate is weak. In contrast, the soft and compressible nanoPTL would create local low pressure regions. Moreover, when the membrane swells during operation, it may favor moving into the softer nanoPTL surface rather than pressing against the IrO_2_ layer, reducing catalyst delamination and transfer onto the membrane.

**Figure 6:**
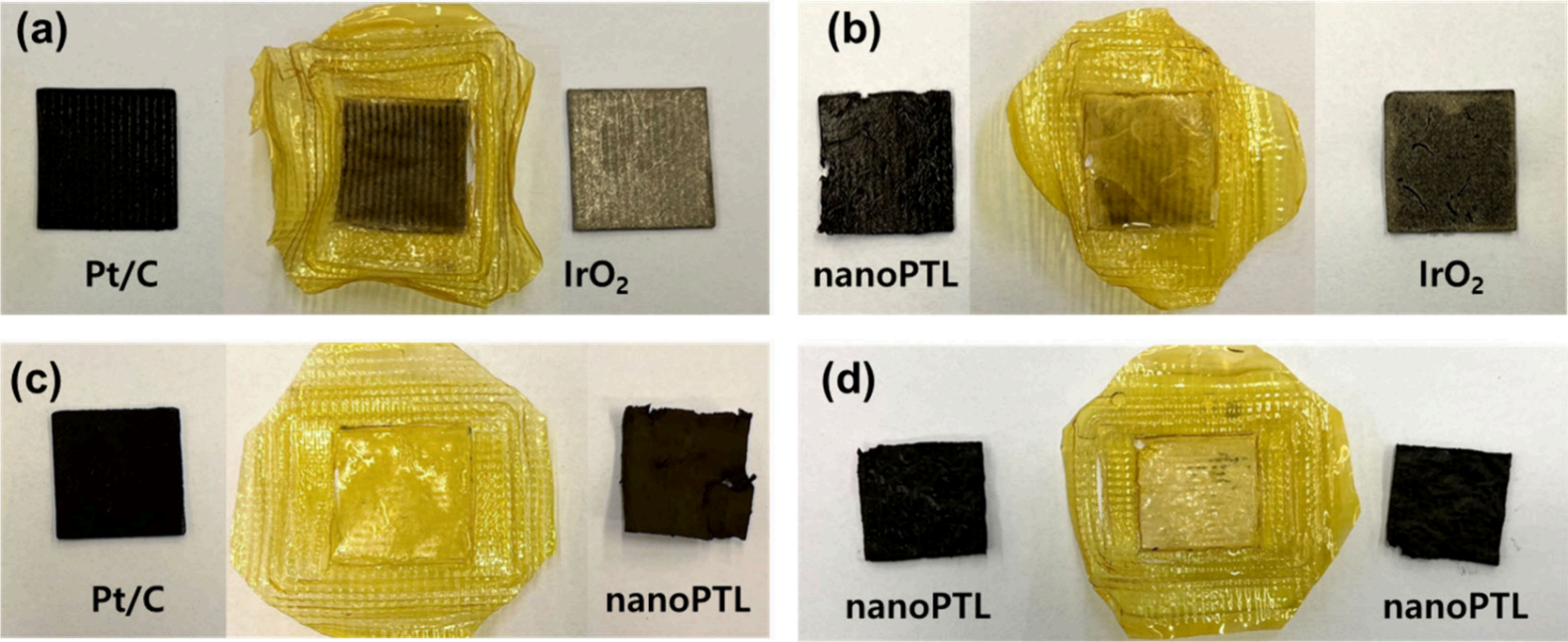
MEAs arranged in the order of cathode, membrane, and anode post operation with (a) Pt/C-IrO_2_ benchmark, (b) nanoPTL-IrO_2_, (c) Pt/C-nanoPTL, and (d) nanoPTL-nanoPTL cells.

The Pt/C-nanoPTL cell and nanoPTL full cell where nanoPTL was used as the anode did not show any visible signs of catalyst delamination or transfer onto the membrane, validating the physical and chemical stabilities of the nanoPTL as a real cell component. These results collectively demonstrate that while the nanoPTL offers sustainable, cost-effective, and material-efficient benefits, it faces some critical issues that need mitigating. Mass transport issues must be addressed when it is used as the cathode by tuning the porosity of the electrode, such as selective pathway drilling or template assisted electrode synthesis. When used as the anode, nanowire-to-nanowire conductivity must be strengthened by welding or sintering post processes, and the intrinsic catalytic activity must be enhanced through the integration of secondary or tertiary elements to compete with the commercial benchmark cell performance.

## 4 Conclusions

In this study, we developed a self-standing, bifunctional catalytic nanoPTL for AEM water electrolysis using a magnetically assisted chemical reduction method. Fine-tuning of the nanowire morphology was achieved by varying the seed type and concentration, where silver was identified as both a more cost-effective and more reactive alternative to platinum. The identified optimal concentration of Ag in the reaction medium as a seed element was 0.4 mol %, yielding a nanoPTL with a 6-fold increase in ECSA and a 90% reduction in Ni loading mass relative to commercial Ni felt PTL. The nanoPTL exhibited exceptional performance in the HER, achieving 1900 mA cm^–2^ at −0.22 V vs RHE, which is a 6-fold higher performance than that of the PtC/GDL benchmark at the same applied potential. It also demonstrated promising OER activity in ex situ tests, though it fell behind a little compared to the IrO_2_/Ni felt benchmark, due to the intrinsic limitations of Ni for the OER and its unwelded nanowire architecture that may introduce contact resistance during anodic operations.

Single-cell tests validated the stable operation, mechanical robustness, and catalytic activity of the nanoPTL in a real operation environment. Importantly, the nanoPTL maintained mechanical robustness under real operating conditions with minimal catalyst delamination. This study also uncovered performance limitations in real cell environments and their likely cause, namely, mass transport constraints when it acts as a cathode, stemming from its fine porous structure. These findings underscore the importance of morphology control not only for catalytic activity but also for gas and water transport efficiency in full-cell configurations.

Overall, the nanoPTL presents a compelling pathway toward simplified, nickel-efficient electrode design for sustainable AEMWE. Future work will focus on optimizing porosity for cathodic applications, enhancing nanowire conductivity via sintering or welding processes, and incorporating secondary catalytic elements to further boost the OER activity. These efforts aim to bridge the gap between material innovation and the commercial viability of AEMWE.

## Supplementary Material



## Notes

Details about sample preparation, material confirmation by XPS depth profiling, SEM characterization, HR-TEM characterization, EDS mapping, BET analysis, and voltage breakdown analysis (PDF)
