# Supplementary material for: Toward Simplified Electrode Design: Development of Nickel-Efficient Catalytic NanoPTL for Sustainable AEM Water Electrolysis
Source: ACS Nanosci Au. 2026 Mar 18;6(3):369–78. doi: 10.1021/acsnanoscienceau.5c00168 (PMC13281195; doi:10.1021/acsnanoscienceau.5c00168)
Supplement: Supplementary file 1 [file ng5c00168_si_001.pdf]

Supporting Information

Toward Simplified Electrode Design: Development of Nickel-Efficient Catalytic NanoPTL for Sustainable AEM Water Electrolysis

Seungyoung Park[c], Seulgi Ji[b], Sara Andrenacci[a], Sun Sook Lee[b], Yejung Choi[a*]*

[a]Kyonggi University, School of Electronic Engineering, Suwon 16227, Republic of Korea

[b]Thin Film Research Center, Korea Research Institute of Chemical Technology, Yuseong, Post Office Box 107, Daejeon 34114, Republic of Korea

[c]Department of Sustainable Energy Technology, SINTEF Industry, 7034 Trondheim, Norway

Corresponding Author

E-mail: yejung.choi@sintef.no (Y. Choi), sunsukl@krict.re.kr (S.S. Lee)

KEYWORDS

porous transport layer, bifunctional catalyst, nickel electrode, AEMWE, magnetically chemical reduction

CONTENTS

Photograph of nanoPTLs seeded with varying amounts of Pt and Ag (Figure S1) ------------- S3

SEM images of Ni nanoPTL with Ag molar percentage to Ni ranging from 0 to 0.8 % (Figure S2) -- S3

TEM images of Ni nanoPTL with Ag molar percentage to Ni ranging in 0.1 and 0.4 % (Figure S3) -- S4

Average wire thickness of Ag and Pt seeded nanoPTLs (Table S1) ----------------------------- S4

SEM images of Ni nanoPTL with Pt molar percentage to Ni ranging from 0.1 to 0.8 % (Figure S4) -- S5

Crystalline structure of the spikey surface on Ag and Pt seeded nanoPTL (Figure S5) ------ S5

TEM and EDS images of Ag seeded Ni nanoPTL after 10 minutes of reaction time (Figure S6) -- S6

TEM and EDS images of Ag seeded Ni nanoPTL after 30 minutes of reaction time (Figure S7) -- S7

TEM and EDS images of Pt seeded nanoPTL after 10 min of reaction time (Figure S8) ----- S8

TEM and EDS images of Pt seeded nanoPTL after 30 min of reaction time (Figure S9) ----- S9

(A) XPS depth profiling spectra of non-seeded, Ag- and Pt-seeded Ni nanoPTL, (B) Etching time-dependent Ag and Pt atomic ratio in Ag and Pt seeded nanoPTL (Figure S10) --------- S10

Nitrogen adsorption isotherms of Ag and Pt seeded nanoPTL (Figure S11) ------------------- S11

BET results for surface area, pore volume, and pore size of Ag and Pt seeded nanoPTLs (Table S2) --- S11

Summary of the voltage breakdown analysis (Figure S12) ------------------------------------ S12

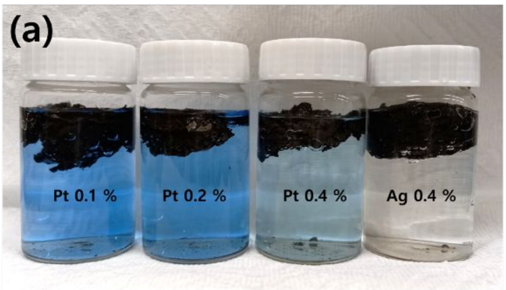

Figure S1. Photograph of nanoPTLs seeded with varying amounts of Pt and Ag.

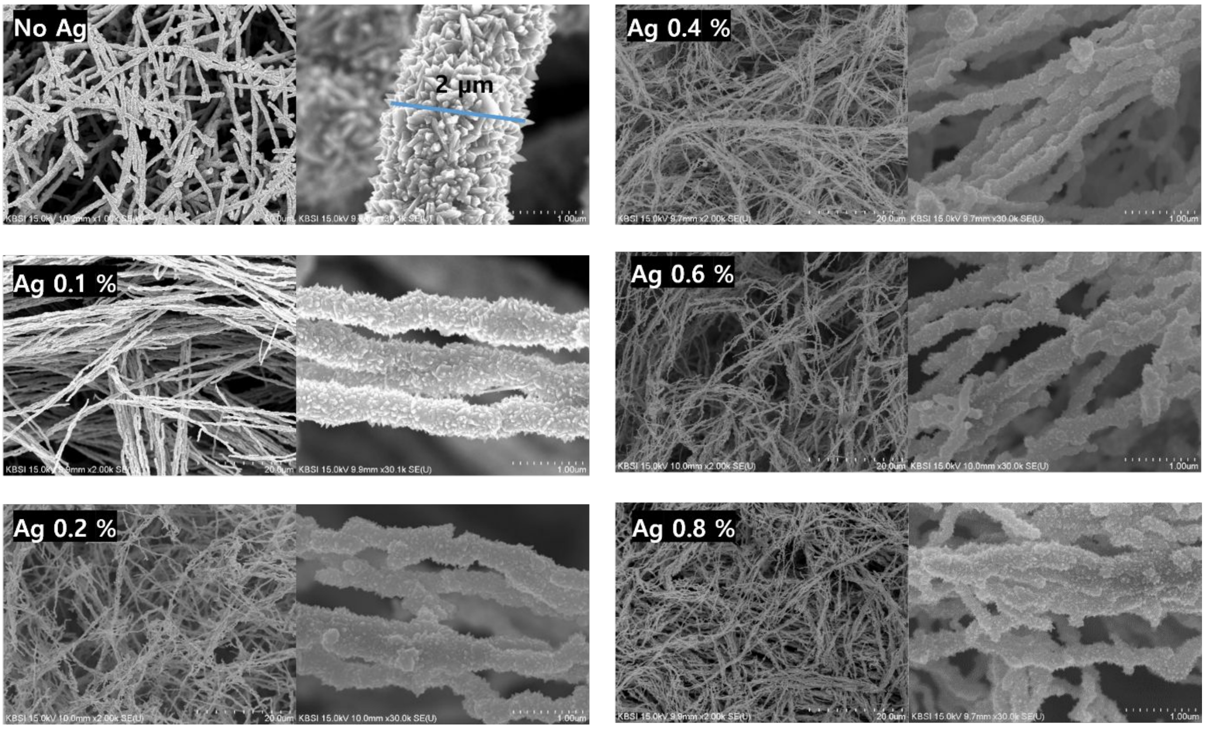

Figure S2. SEM images of Ni nanoPTL with Ag molar percentage to Ni ranging from 0 to 0.8 %.

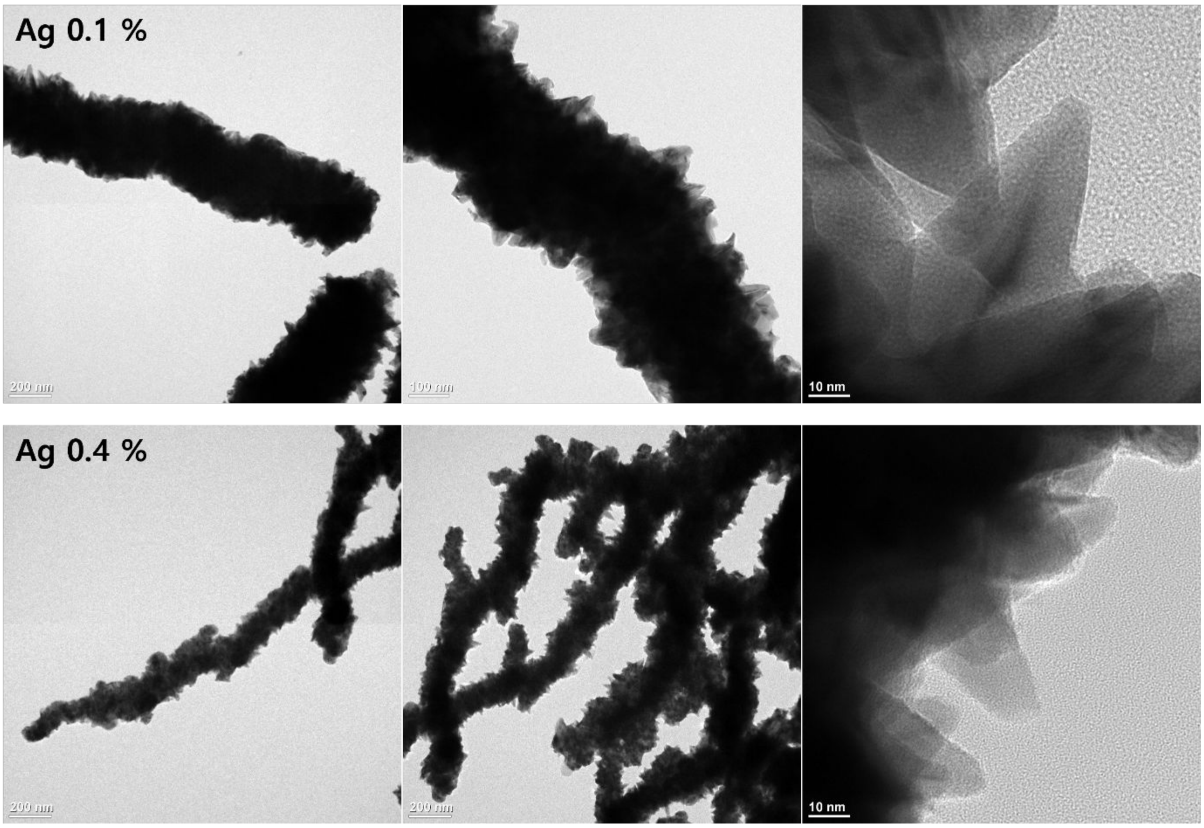

Figure S3. TEM images of Ni nanoPTL with Ag molar percentage to Ni ranging in 0.1 and 0.4 %.

Sample	SIZE (nm)	Sample	SIZE (nm)
Ag or Pt x	1600		
Ag 0.1 mol %	324	Pt 0.1 mol %	410
Ag 0.2 mol %	391	Pt 0.2 mol %	395
Ag 0.4 mol %	131	Pt 0.4 mol %	376
Ag 0.6 mol %	247	Pt 0.6 mol %	393
Ag 0.8 mol %	288	Pt 0.8 mol %	402

Table S1. Average wire thickness of Ag and Pt seeded nanoPTLs.

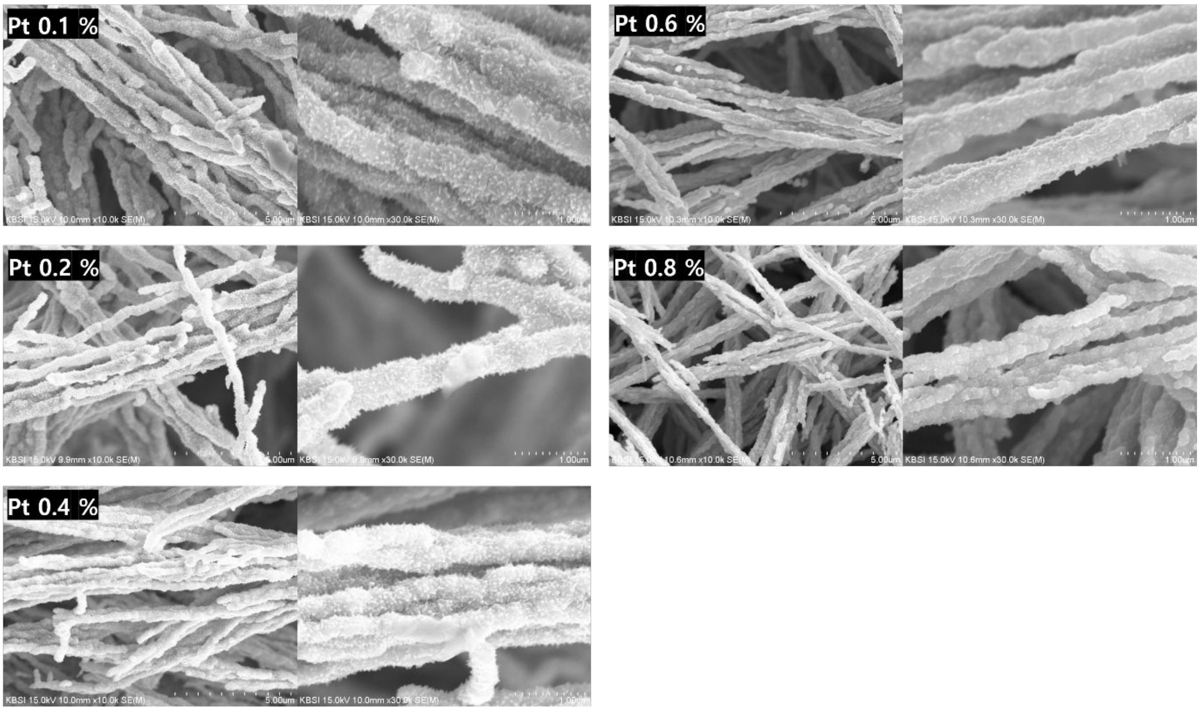

Figure S4. SEM images of Ni nanoPTL with Pt molar percentage to Ni ranging from 0.1 to 0.8 %

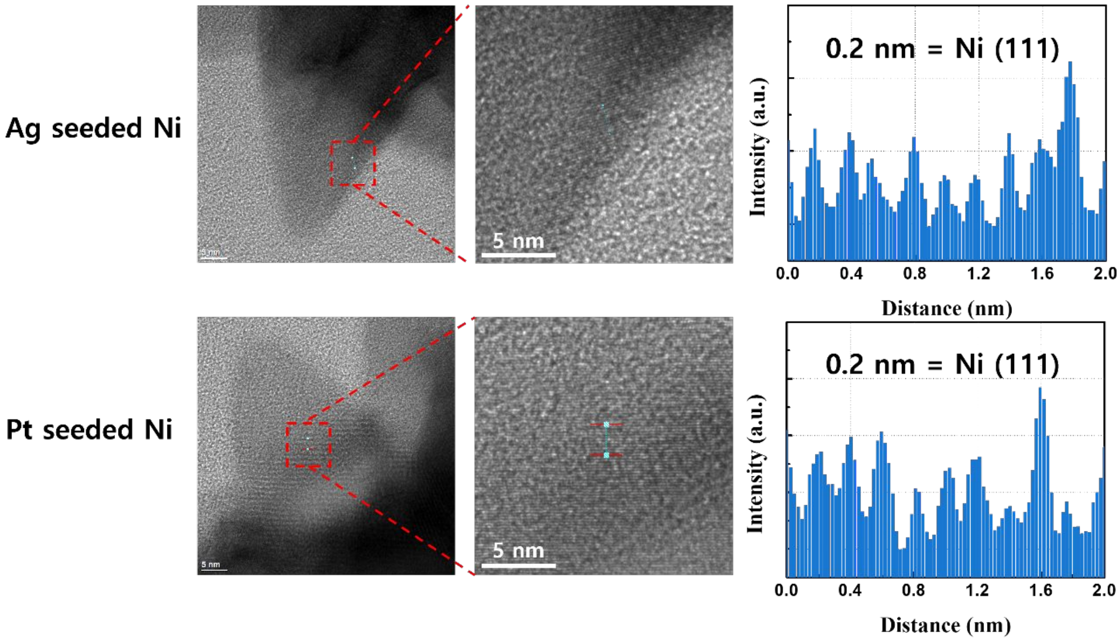

Figure S5. Crystalline structure of the spikey surface on Ag and Pt seeded nanoPTL.

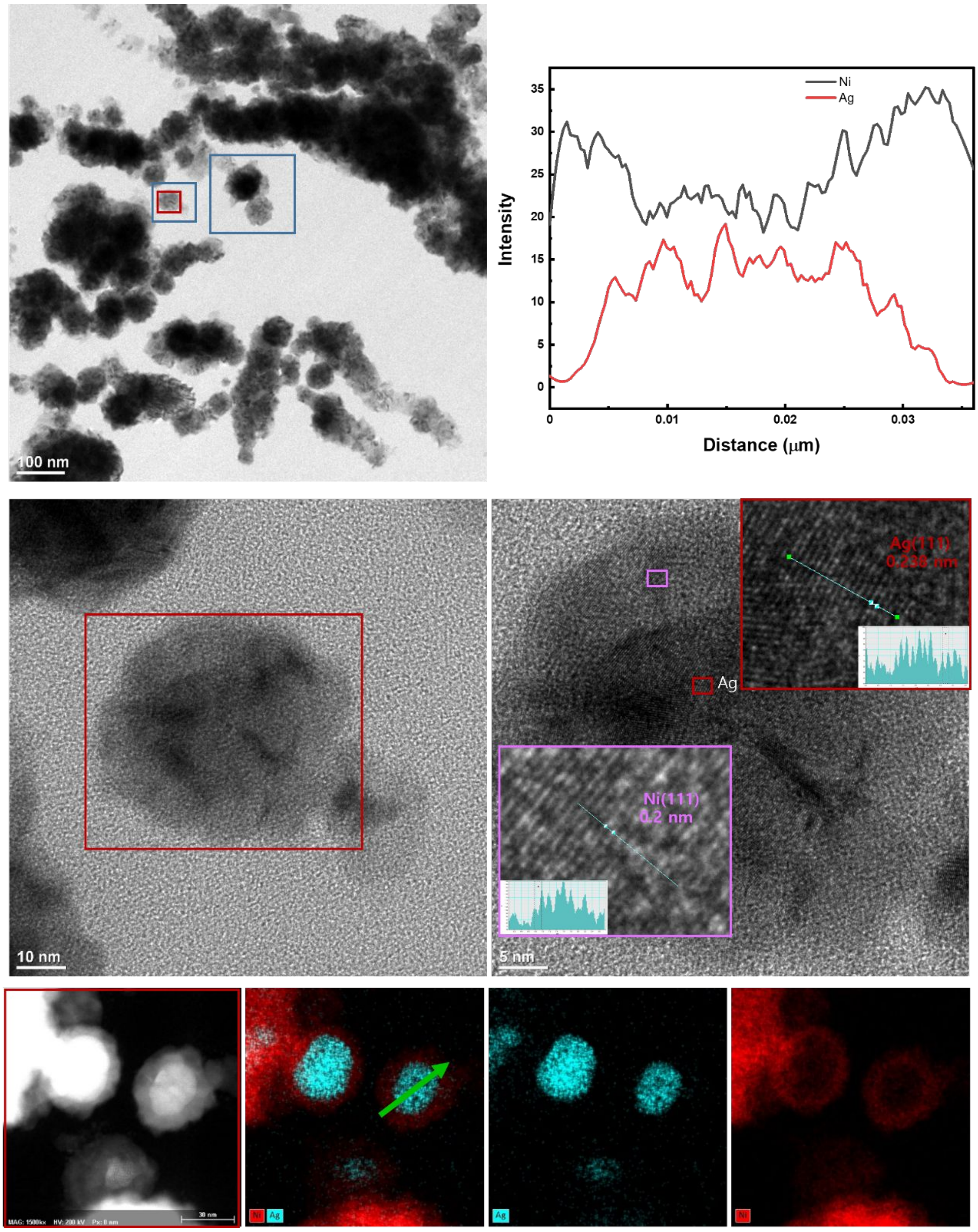

Figure S6. TEM and EDS images of Ag seeded Ni nanoPTL after 10 minutes of reaction time.

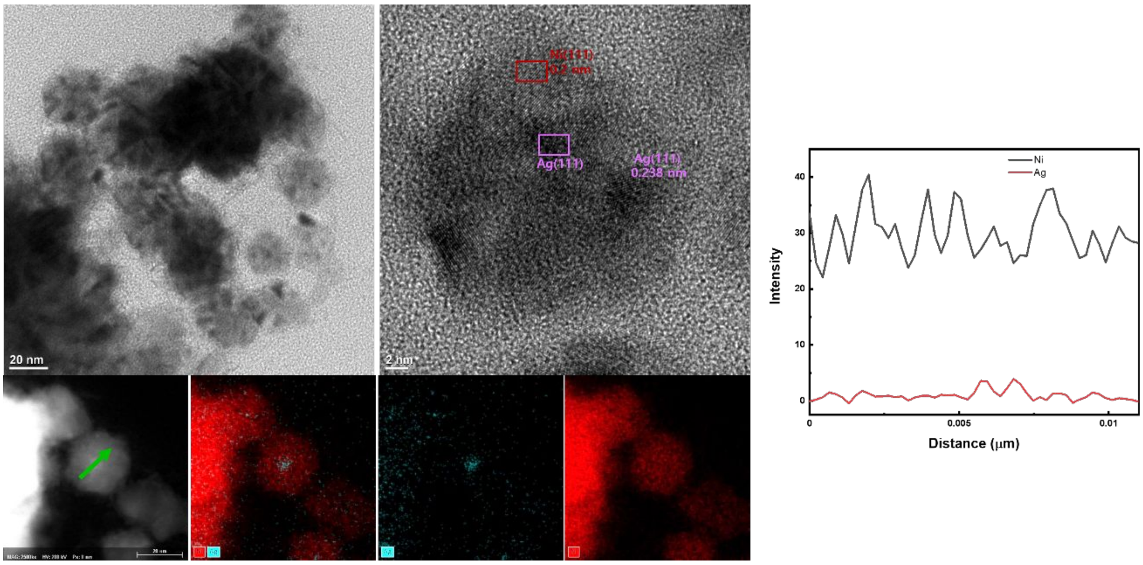

Figure S7. TEM and EDS images of Ag seeded Ni nanoPTL after 30 minutes of reaction time.

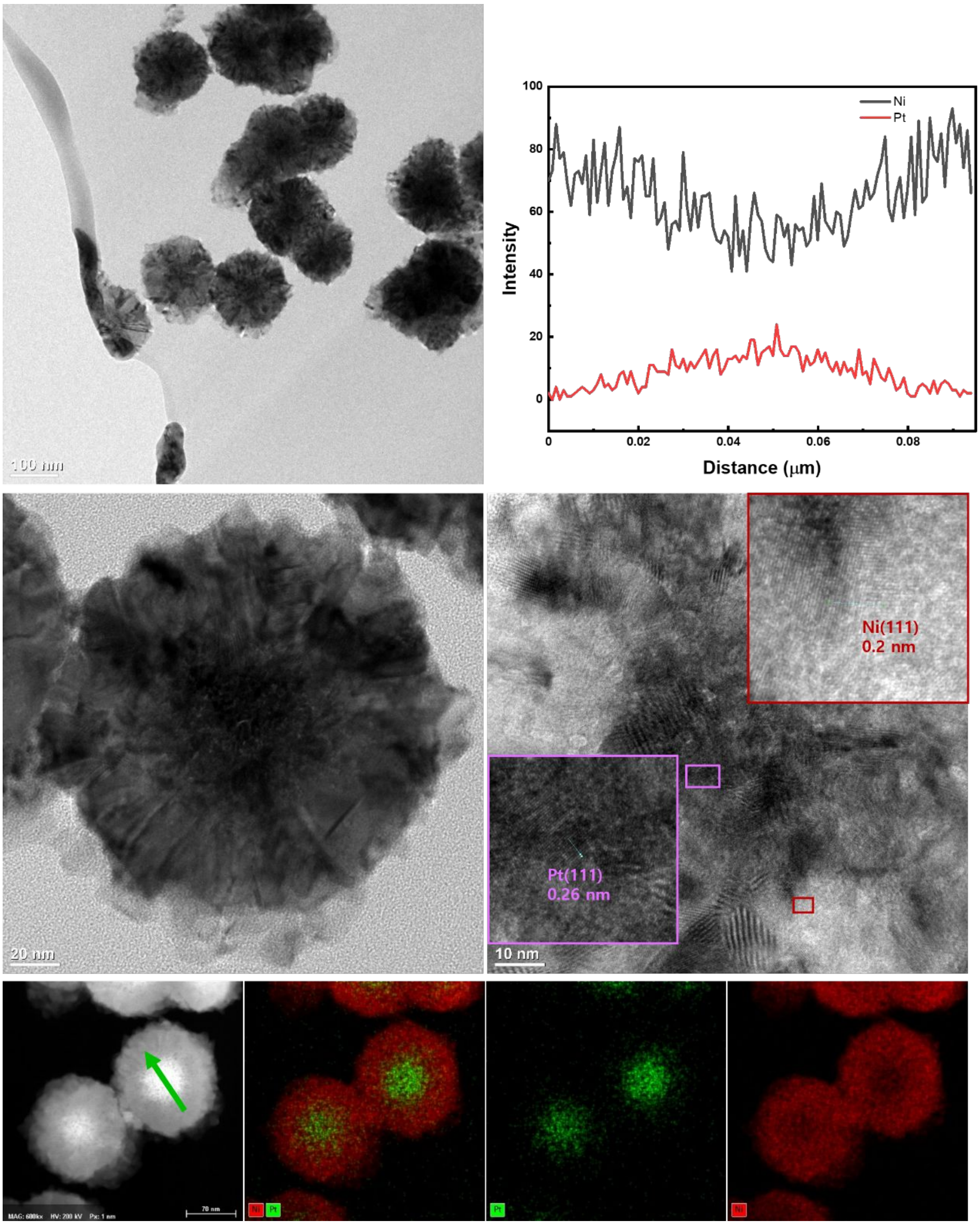

Figure S8. TEM and EDS images of Pt seeded Ni nanoPTL after 10 minutes of reaction time.

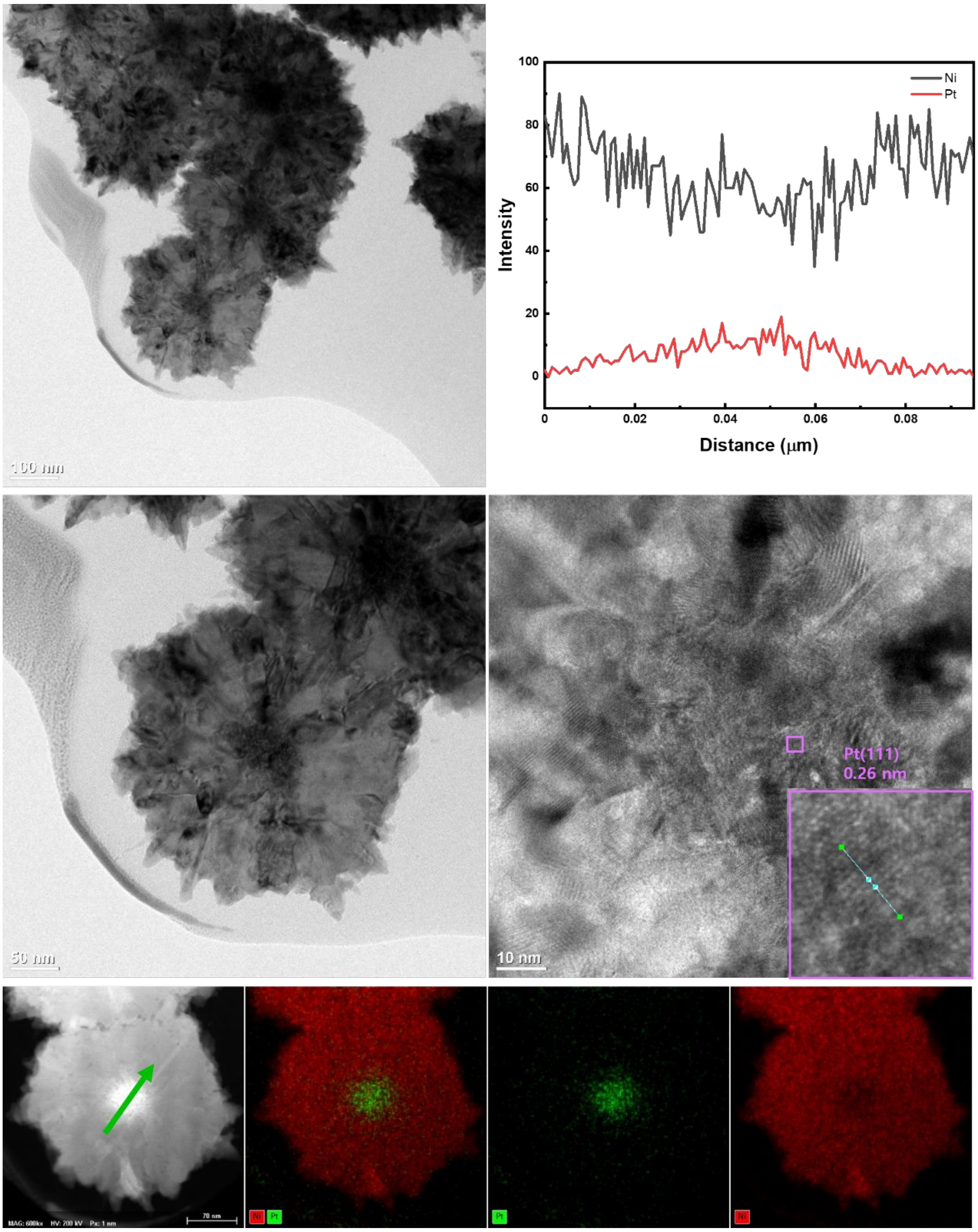

Figure S9. TEM and EDS images of Pt seeded Ni nanoPTL after 30 minutes of reaction time.

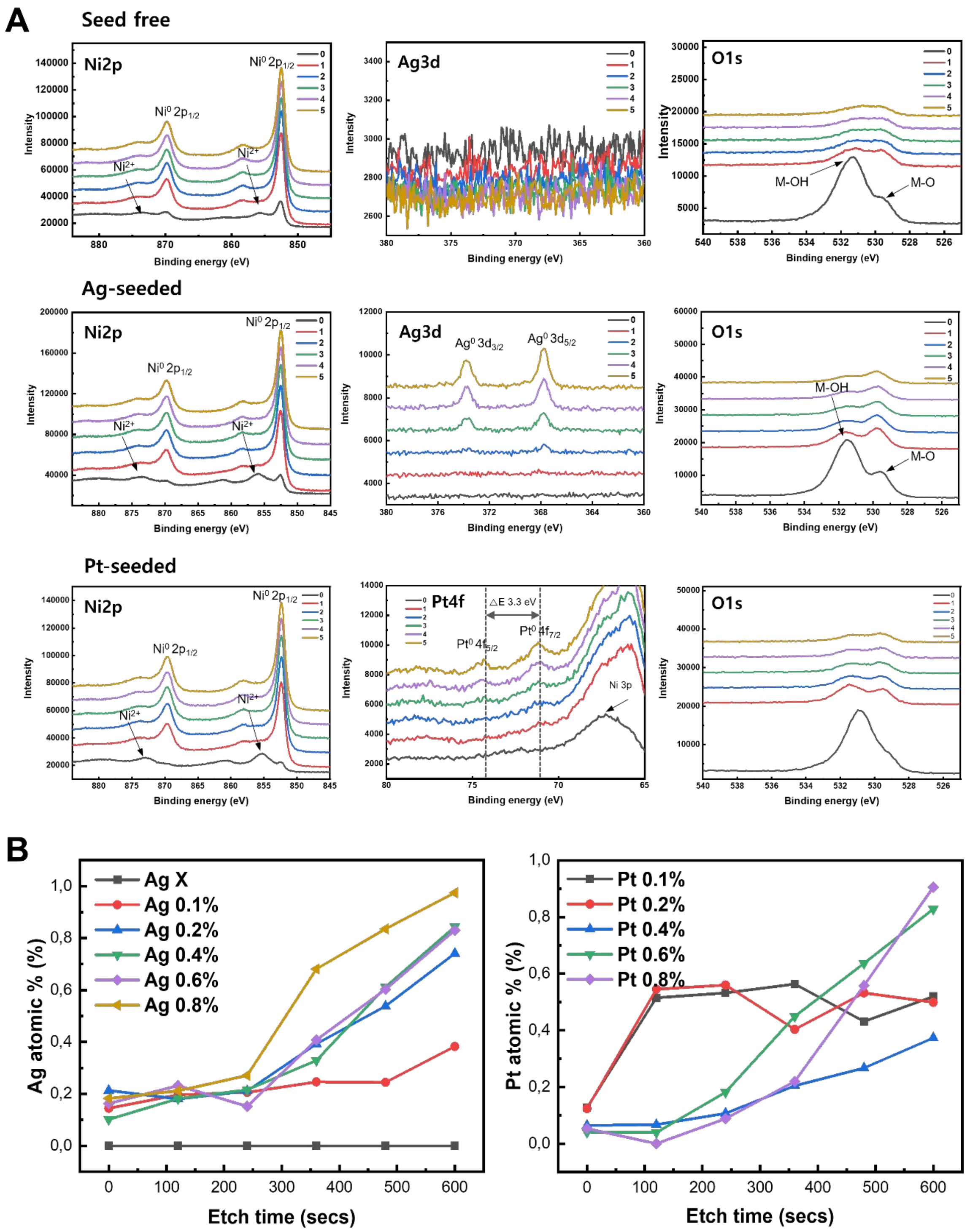

Figure S10. (A) XPS depth profiling spectra of non-seeded, Ag-seeded, and Pt-seeded Ni nanoPTL, (B) Etching time-dependent Ag and Pt atomic ratio in Ag and Pt seeded nanoPTL.

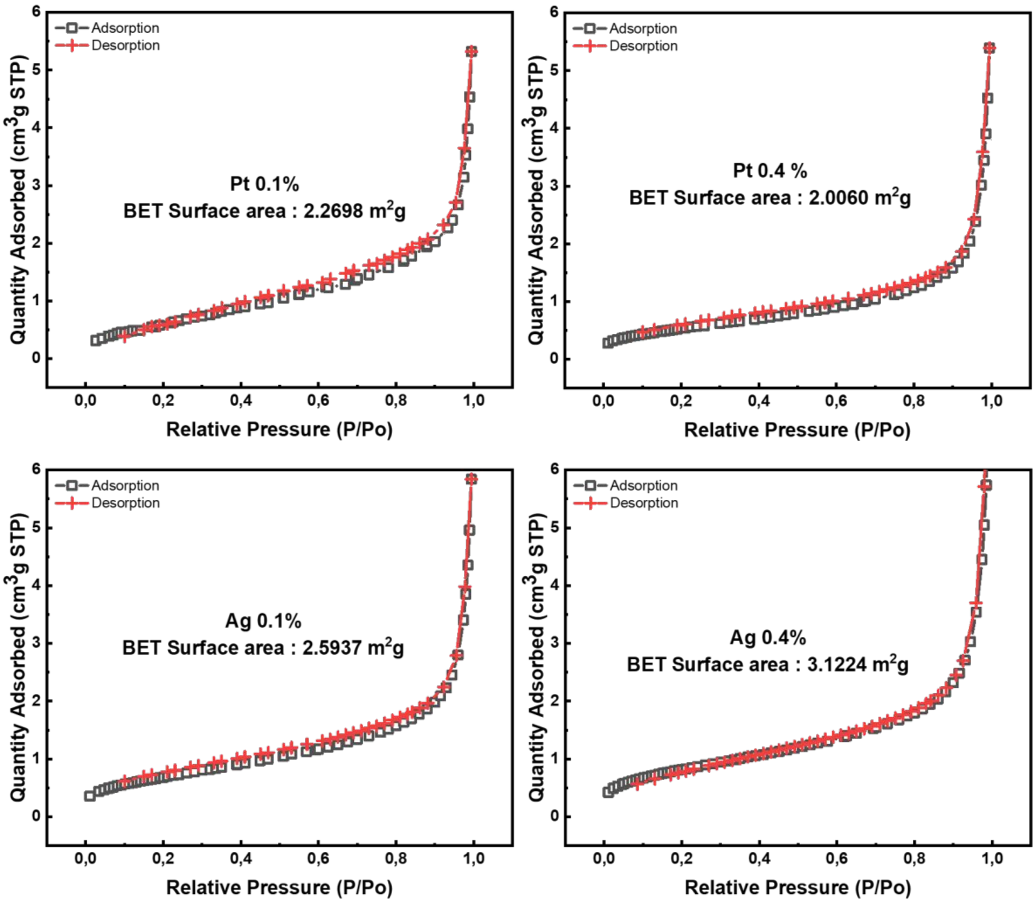

Figure S11. Nitrogen adsorption isotherms of Ag and Pt seeded nanoPTL

	Pt 0.1%	Pt 0.4%	Ag 0.1%	Ag 0.4%
BET Surface area	2.2698 m^2/g	2.0060 m^2/g	2.5542 m^2/g	3.1224 m^2/g
Pore Volume	0.008227 cm^3/g	0.008341 cm^3/g	0.009034 cm^3/g	0.012377 cm^3/g
Pore size	144.9810 Å	166.3085 Å	141.4798 Å	158.5578 Å

Table S2. BET results for surface area, pore volume, and pore size of Ag and Pt seeded nanoPTLs.

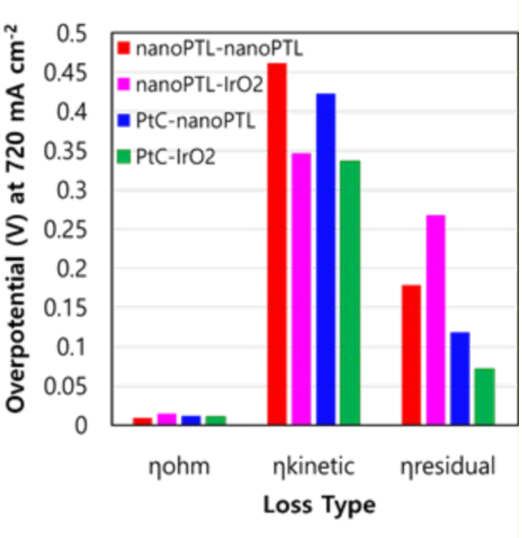

Figure S12. Summary of the voltage breakdown analysis at 720 mA/cm^2 for the tested cell configurations.
